# Nuclear Factor-erythroid 2 (NF-E2) p45-related Factor-2 (Nrf2) Modulates Dendritic Cell Immune Function through Regulation of p38 MAPK-cAMP-responsive Element Binding Protein/Activating Transcription Factor 1 Signaling*

**DOI:** 10.1074/jbc.M113.483420

**Published:** 2013-06-17

**Authors:** Laith M. A. Al-Huseini, Han Xian Aw Yeang, Swaminathan Sethu, Naif Alhumeed, Junnat M. Hamdam, Yulia Tingle, Laiche Djouhri, Neil Kitteringham, B. Kevin Park, Christopher E. Goldring, Jean G. Sathish

**Affiliations:** From the ‡ Medical Research Council Centre for Drug Safety Science and Department of Molecular and Clinical Pharmacology, Sherrington Buildings, Ashton Street, University of Liverpool, Liverpool L69 3GE, United Kingdom and; § Department of Pharmacology and Therapeutics, College of Medicine, Al-Qadisiyah University, Diwaniyah, P.O. BOX 80, Iraq

**Keywords:** Dendritic Cells, Heme Oxygenase, Nrf2, p38 MAPK, Reactive Oxygen Species (ROS), CREB/ATF1, Co-stimulation

## Abstract

Nrf2 is a redox-responsive transcription factor that has been implicated in the regulation of DC immune function. Loss of Nrf2 results in increased co-stimulatory molecule expression, enhanced T cell stimulatory capacity, and increased reactive oxygen species (ROS) levels in murine immature DCs (iDCs). It is unknown whether altered immune function of Nrf2-deficient DCs (Nrf2^−/−^ iDCs) is due to elevated ROS levels. Furthermore, it is unclear which intracellular signaling pathways are involved in Nrf2-mediated regulation of DC function. Using antioxidant vitamins to reset ROS levels in Nrf2^−/−^ iDCs, we show that elevated ROS is not responsible for the altered phenotype and function of these DCs. Pharmacological inhibitors were used to explore the role of key MAPKs in mediating the altered phenotype and function in Nrf2^−/−^ iDCs. We demonstrate that the increased co-stimulatory molecule expression (MHC II and CD86) and antigen-specific T cell activation capacity observed in Nrf2^−/−^ iDCs was reversed by inhibition of p38 MAPK but not JNK. Importantly, we provide evidence for increased phosphorylation of cAMP-responsive element binding protein (CREB) and activating transcription factor 1 (ATF1), transcription factors that are downstream of p38 MAPK. The increased phosphorylation of CREB/ATF1 in Nrf2^−/−^ iDCs was sensitive to p38 MAPK inhibition. We also show data to implicate heme oxygenase-1 as a potential molecular link between Nrf2 and CREB/ATF1. These results indicate that dysregulation of p38 MAPK-CREB/ATF1 signaling axis underlies the altered function and phenotype in Nrf2-deficient DCs. Our findings provide new insights into the mechanisms by which Nrf2 mediates regulation of DC function.

## Teaser

**Background:** Nrf2 is required for normal dendritic cell immune functions.

**Results:** Loss of Nrf2 alters DC function and results in hyperphosphorylation of CREB/ATF1 transcription factors that are responsive to p38 MAPK inhibition.

**Conclusion:** The p38 MAPK-CREB/ATF1 axis contributes to Nrf2-mediated regulation of DC function.

**Significance:** Defining the relevance of p38-CREB/ATF1 in Nrf2 signaling expands understanding of DC biology.

## Introduction

Dendritic cells (DCs)^3^ are antigen-presenting cells pivotal for the induction of primary adaptive immune responses. Immature DCs (iDCs) express low levels of MHC II and other co-stimulatory molecules such as CD80, CD86, and CD40, with limited capacity to induce antigen-specific T-cell activation. DC maturation is associated with up-regulation of co-stimulatory molecules and cytokine production, renders the DCs competent in T cell activation and elicitation of an immune response (1). The MAPKs represent vital intracellular signaling pathways that regulate a variety of cellular processes, including cell differentiation, proliferation, and apoptosis. There are at least three distinct MAPK pathways in mammals, including extracellular signal-regulated 1/2 kinases (ERK1/2), JNK, and p38 MAPK (2, 3). MAPKs activation is important for regulation of DC maturation, survival, and cytokine secretion (4). Importantly, the p38 MAPK pathway has been shown to regulate DC co-stimulatory receptor expression, T cell proliferative capabilities, and cytokine production (5, 6). Signaling through the p38 MAPK pathway results in the downstream activation of cAMP-responsive element binding protein (CREB) and activating transcription factor 1 (ATF1) (7). Phosphorylation of CREB is known to be associated with up-regulation of CD86 and secretion of the cytokine, IL-10 (8, 9).

Redox homeostasis is important for a variety of cellular functions such as proliferation, apoptosis, and intracellular signaling pathways, including MAPK signaling (10–12). In the context of DCs, alterations in cellular reactive oxygen species (ROS) and redox status result in changes in immune function such as maturation and cytokine production, which subsequently impacts on the type of T cell immune response elicited (13, 14). Nuclear factor erythroid 2-related factor 2 (Nrf2) is a redox-sensitive, basic-leucine zipper transcription factor (15, 16). Nrf2 is expressed in a variety of cell types, including DCs, where it contributes to maintenance of redox homeostasis (17, 18) by regulating key cytoprotective/antioxidant genes, including glutathione (GSH), heme oxygenase-1 (HO-1), NAD(P)H:quinine oxidoreductase 1, and superoxide dismutases (19). Hemo-oxygenase-1 is a rate-limiting enzyme in the catabolism of heme and exhibits antioxidant, anti-inflammatory, anti-apoptotic and immunomodulatory properties, and has been implicated in DC differentiation and maturation (20–25).

We and others (14, 26) have shown that loss of Nrf2 in iDCs results in increased intracellular ROS, enhanced co-stimulatory molecules expression, impaired antigen capture capacity, and enhanced capacity for antigen-specific CD8 T cell stimulation. However, it is not known whether the elevated ROS level, in the absence of Nrf2, is responsible for the changes in the DC immune function. Furthermore, it is not clear what signaling pathways are involved in mediating the changes observed in the absence of Nrf2. In this study, using Nrf2-deficient iDCs, we demonstrate that elevated ROS levels do not underlie altered immune function in these DCs. We also show that functional changes associated with the loss of Nrf2 in iDCs is sensitive to pharmacological inhibition of p38 MAPK but not JNK activity. Importantly, we demonstrate that CREB and ATF1 are hyperphosphorylated in the absence of Nrf2. Our results also show that CREB and ATF1 hyperphosphorylation can be induced through inhibition of HO-1 activity. Our findings highlight the importance of the p38 MAPK-CREB signaling axis in Nrf2-mediated regulation of DC immune function.

## EXPERIMENTAL PROCEDURES

### 

#### 

##### Reagents

All reagents were from Sigma-Aldrich unless otherwise stated. FCS (Invitrogen), SB203580 and PD98059 (Cell Signaling Technology, Danvers, MA), and tin protoporphyrin IX dichloride (Tocris Bioscience, Bristol, UK) were also purchased for the study.

##### Mice

Nrf2^+/+^ and Nrf2^−/−^ mice were purchased from Riken BioResource Center (Ibaraki, Japan) and maintained at the Biomedical Services Unit, University of Liverpool (27). Mice transgenic for the H-2D^b^-restricted TCR-αβ transgene, F5, were a kind gift from Dr. James Matthews (Cardiff, Wales, UK). Protocols described herein were undertaken in accordance with criteria outlined in license granted under the Animals (Scientific Procedures) Act 1986 (PPL 40/3379).

##### Generation of Bone Marrow-derived DCs

Bone marrow-derived iDCs were generated from Nrf2^+/+^ and Nrf2^−/−^ mice according to published protocol (28). On day 6 cells were harvested, and CD11c-positive DCs were isolated using magnetic beads by positive selection according to the manufacturer's instructions (MiltenyiBiotec, Surrey, UK). The purity of the isolated DC population was >90% as determined by flow cytometry on a BD FACSCanto II flow cytometer (BD Biosciences, Oxford, UK).

##### Cell Surface Receptor Expression

DCs were stained with fluorescent αCD11c^TC^ (Invitrogen) and αCD86^FITC^, or αMHCII^PE^ (BD Biosciences) antibodies for 30 min on ice, washed, acquired on a BD FACSCanto II flow cytometer (BD Biosciences), and analyzed using Cyflogic software (version 1.2.1, CyFlo, Ltd.).

##### Measurement of ROS

Basal or vitamin-treated iDCs from Nrf2^+/+^ and Nrf2^−/−^ mice were stained using fluorescent ROS indicator, dihydroethidium, according to Ref. 29, and analyzed by flow cytometry.

##### F5 CD8 T Cell Proliferation

F5 CD8 T cell proliferation was quantified as described previously (30). Briefly, Nrf2^+/+^ and Nrf2^−/−^ iDCs were pulsed with a dose range of antigenic peptide (NP68), washed, and co-cultured with F5 CD8 T cells for 72 h. [^3^H]Thymidine was added for the last 16 h. Cells were harvested onto glass fiber filter mats and read on a scintillation counter (MicroBetaTrilux; PerkinElmer Life Sciences, Buckinghamshire, UK).

##### Gel Electrophoresis and Western Immunoblotting

Nrf2^+/+^ and Nrf2^−/−^ iDCs were lysed, and 20 μg of lysate protein was resolved by SDS-PAGE, transferred to nitrocellulose membranes (GE Healthcare), blocked, and probed for the indicated proteins using the appropriate primary antibodies; phospho-p38, p38, phospho-CREB, CREB (Cell Signaling Technology, Danvers, MA); and α-tubulin (Santa Cruz Biotechnology) followed by horseradish peroxidase-conjugated secondary antibodies (Cell Signaling Technology) and visualized using the ECL system (PerkinElmer Life Sciences).

##### ELISA

Cell-free culture supernatants were used for measuring IL-10 cytokine concentrations by sandwich ELISA (Quantikine, R&D Systems, Abingdon, UK). On day 6, DCs were seeded onto 24-well plates in duplicates at a density of 8 × 10^5^ DCs per ml at 2 ml/well. After 48 h, cell-free supernatants from SB203580 untreated and treated groups with or without the Toll-like receptor agonist LPS were collected and stored at −20 °C until further analysis.

##### Statistics

Raw data obtained were analyzed using the unpaired *t* test or one-way ANOVA. *p* values < 0.05 were considered to be statistically significant.

## RESULTS

### 

#### 

##### Altered Immature DC Function Due to the Loss of Nrf2 Is Not Dependent on Elevated ROS

**FIGURE 1.**
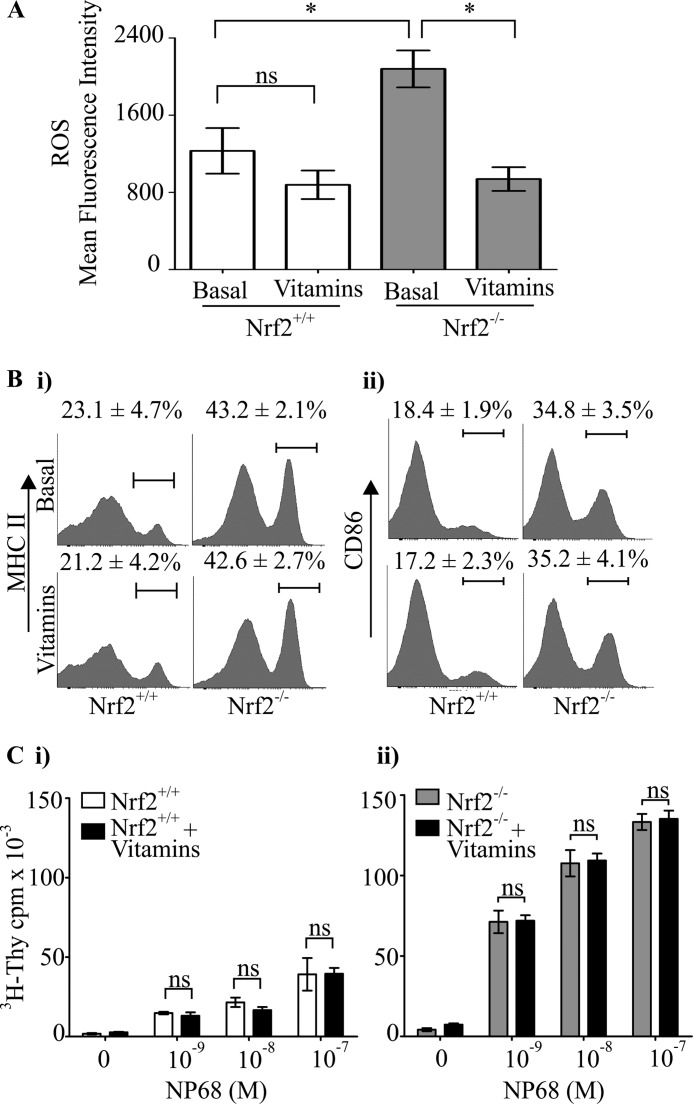
**Reducing ROS levels does not restore altered phenotype and function of Nrf2^−/−^ iDCs.** Nrf2^+/+^ and Nrf2^−/−^ iDCs were treated with or without vitamins C (1 mm) and E (100 μm) for 48 h. *A*, cells were incubated with the ROS indicator dihydroethidium and analyzed by flow cytometry. Data are presented as average mean fluorescence intensity ± S.D. and derived from three independent experiments. *B*, DCs were labeled with fluorescent conjugated antibodies against MHC II and CD86 co-stimulatory molecules. Co-stimulatory molecule expression was determined by flow cytometry. The percentages of iDCs expressing high levels MHC II **(***i***)** and CD86 **(***ii***)** are indicated *above* the marker. Representative histograms are presented with average percentage ± S.D. Data are derived from three independent experiments. *C*, Nrf2^+/+^ (*i*) or Nrf2^−/−^ (*ii*) iDCs were pulsed with increasing concentrations of NP68 antigenic peptide and then co-cultured with F5 CD8 T cells for 72 h. [^3^H]Thymidine (^3^H-Thy) was added for the last 16 h. Proliferation of T cells was determined by scintillation counting of incorporated [^3^H]thymidine. Data are presented as average [^3^H]thymidine scintillation counts ± S.D. Statistical significance was assessed using unpaired Student's *t* test or one-way ANOVA. Data are representative of three independent experiments (*, *p* < 0.05; *NS*, not significant).

Loss of Nrf2 leads to increased co-stimulatory molecule expression, T cell stimulatory potential, and elevated ROS levels in iDCs (14, 26). We investigated whether the elevated ROS contributed to increased co-stimulatory molecules expression by reducing ROS to normal levels using antioxidants in these cells. Vitamins C and E have antioxidant activity and are known to reduce ROS levels (31, 32). Nrf2^+/+^ and Nrf2^−/−^ iDCs were treated with vitamins C and E for 48 h, and ROS levels measured by flow cytometry using the fluorescent ROS indicator dihydroethidium. A significant reduction in ROS levels was observed in vitamin-treated Nrf2^−/−^ iDCs compared with untreated controls (mean fluorescence intensity, 2079 *versus* 938, *p* < 0.05) as shown in Fig. 1*A*. A slight reduction in the ROS levels was also seen in vitamin-treated Nrf2^+/+^ iDCs, which was not statistically significant (mean fluorescence intensity 1230 *versus* 878, *p* > 0.05) (Fig. 1*A*). It is important to note that the ROS levels in vitamin-treated Nrf2^−/−^ iDCs was equivalent to that in untreated Nrf2^+/+^ iDCs (Fig. 1*A*). To test whether elevated intracellular redox levels is responsible for increased DC co-stimulatory molecule expression, we measured MHC II and CD86 expression in Nrf2^−/−^ iDCs following vitamins treatment. As shown in Fig. 1*B*, the expression of MHC II and CD86 under basal condition was significantly higher in the Nrf2^−/−^ iDCs compared with Nrf2^+/+^ iDCs (MHC II 43.2% *versus* 23.1%, *p* < 0.05; CD86 34.8% *versus* 18.4%, *p* < 0.05). However, there was no significant difference in the co-stimulatory molecules expression between untreated controls and vitamins treatment groups in both Nrf2^+/+^ (MHC II 23.1% *versus* 21.2% *p* > 0.05; CD86 18.4% *versus* 17.2%, *p* > 0.05) and Nrf2^−/−^ iDCs (MHC II 43.2% *versus* 42.6% *p* > 0.05; CD86 34.8% *versus* 35.2%, *p* > 0.05). This result indicates that restoring ROS levels in Nrf2^−/−^ to Nrf2^+/+^ status did not reverse co-stimulatory molecule expression. We further investigated whether the lack of change in co-stimulatory molecule expression in Nrf2^−/−^ iDCs to ROS reset is also reflected in its ability to induce antigen-specific T cell activation. To determine this, we utilized a TCR transgenic mouse model wherein the CD8 T cells express a T cell receptor (F5 TCR) that responds to an antigenic peptide, NP68, when presented by DCs (33). Using this system, we have previously shown that NP68-bearing Nrf2^−/−^ iDCs stimulated F5 CD8 T cell proliferation more potently than its wild type counterpart (26). Consistent with our previous findings, antigenic peptide-bearing Nrf2^−/−^ iDCs induced higher F5 CD8 T cell proliferation compared with Nrf2^+/+^ iDCs (Fig. 1*C*). Lowering of ROS levels by vitamin treatment did not reduce the potential of NP68-bearing Nrf2^−/−^ iDCs to stimulate F5 CD8 T cell proliferation (Fig. 1*C*). These results demonstrate that altered ROS status associated with loss of Nrf2 does not contribute to increased co-stimulatory molecules expression and T cell stimulatory potential of DCs.

##### Contribution of p38 MAPK Signaling to Nrf2-dependent Regulation of DC Immune Function

**FIGURE 2.**
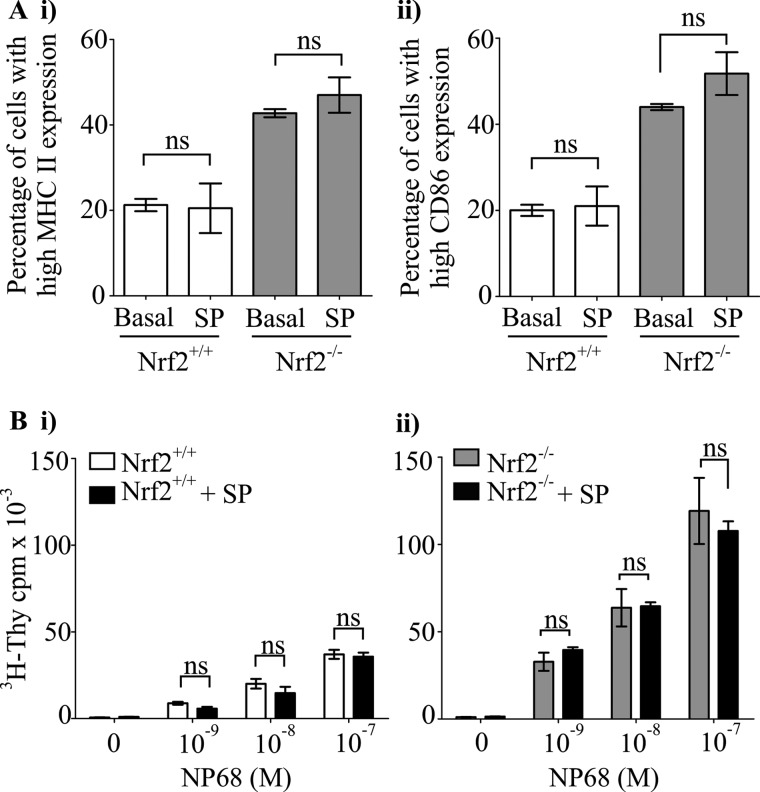
**JNK activity is not required for increased co-stimulatory molecule expression of Nrf2^−/−^ iDCs and T cell activation.** Nrf2^+/+^ and Nrf2^−/−^ iDCs treated with or without 10 μm of JNK inhibitor, SP600125 (*SP*) for 48 h. *A*, MHC II (*i*) and CD86 (*ii*) expression was determined by flow cytometry and presented as percentage of cells expressing high MHC II or CD86. Data derived from three independent experiments are presented as average percentage ± S.D. *B*, Nrf2^+/+^ (*i*) and Nrf2^−/−^ (*ii*) iDCs were pulsed with increasing concentrations of NP68 antigenic peptide and co-cultured with F5 CD8 T cells for 72 h. [^3^H]Thymidine (^3^H-Thy) was added for the last 16 h. Proliferation of T cells was determined by scintillation counting of incorporated [^3^H]thymidine. Data are presented as average [^3^H]thymidine scintillation counts ± S.D. Statistical significance was assessed using unpaired Student's *t* test or one-way ANOVA. Data are representative of three independent experiments (*NS*, not significant).

**FIGURE 3.**
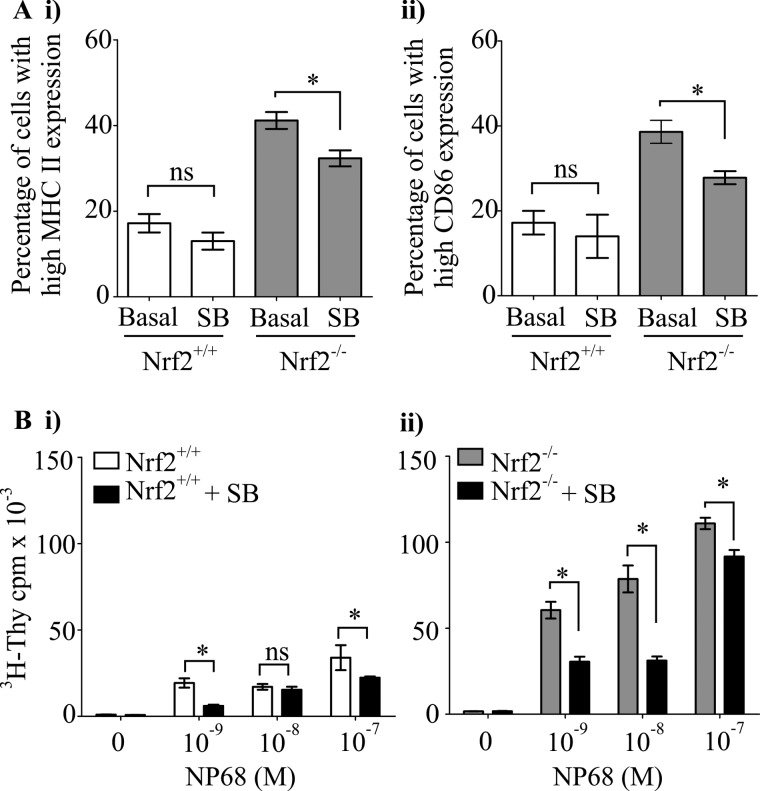
**Contribution of p38 MAPK activity toward altered phenotype and function of Nrf2^−/−^ iDCs.** Nrf2^+/+^ and Nrf2^−/−^ iDCs treated with or without 20 μm of p38 MAPK inhibitor, SB203580 (*SB*) for 48 h. *A*, MHC II (*i*) and CD86 (*ii*) expression was determined by flow cytometry and presented as percentage of cells expressing high MHC II or CD86. Data derived from three independent experiments are presented as average percentage ± S.D. *B*, Nrf2^+/+^ (*i*) and Nrf2^−/−^ (*ii*) iDCs were pulsed with increasing concentrations of NP68 antigenic peptide and co-cultured with F5 CD8 T cells for 72 h. [^3^H]Thymidine (^3^H-Thy) was added for the last 16 h. Proliferation of T cells was determined by scintillation counting of incorporated [^3^H]thymidine. Data are presented as average [^3^H]thymidine scintillation counts ± S.D. Statistical significance was assessed using unpaired Student's *t* test and one-way ANOVA. Data are representative of three independent experiments (*, *p* < 0.05; *NS*, not significant).

Mitogen-activated protein kinase signaling comprising of ERK1/2, p38 MAPK, and JNK pathways coordinate DCs co-stimulatory molecules expression and DC-mediated T cell activation (5, 34, 35). Using pharmacological approaches, we have previously shown that ERK1/2 was not critical in mediating Nrf2-dependent modulation of DC function (26). In the current study, we investigated the role of p38 MAPK and JNK in this context using synthetic inhibitors of JNK and p38 MAPK (JNK-specific inhibitor, SP600125; p38 activity inhibitor, SB203580). JNK inhibition did not reverse the enhanced co-stimulatory molecules expression in Nrf2^−/−^ iDCs to that of the wild type levels (MHC II, 42.8% *versus* 47.0%; *p* > 0.05; CD86, 44.0% *versus* 51.8%; *p* > 0.05 in Nrf2^−/−^ iDCs; and MHC II, 21.3% *versus* 20.5%, *p* > 0.05; CD86, 20.0% *versus* 21.0%, *p* > 0.05 in Nrf2^+/+^ iDCs) as indicated in Fig. 2*A*. The iDC-mediated antigen-specific CD8 T cell proliferation also remained unaltered following JNK inhibition in Nrf2^−/−^ and Nrf2^+/+^ iDCs (Fig. 2*B*). However, inhibition of p38 MAPK activity caused a significant reduction in co-stimulatory molecule expression in Nrf2^−/−^ iDCs (Fig. 3*A*) (MHC II, 41.0% *versus* 32.3%, *p* < 0.05; CD86, 38.6% *versus* 27.8%, *p* < 0.05). Inhibition of p38 MAPK in Nrf2^+/+^ iDCs resulted in only a slight, statistically insignificant reduction in co-stimulatory molecules expression (MHC II, 17.2% *versus* 13.0%, *p* > 0.05; CD86, 17.2% *versus* 14.0%, *p* > 0.05). Consistent with the changes in co-stimulatory molecule expression, inhibition of p38 MAPK resulted in significant reductions in DC-mediated antigen-specific CD8 T cell proliferation in Nrf2^−/−^ iDCs with less pronounced effects on Nrf2^+/+^ iDCs (Fig. 3*B*). These observations suggest the contribution of p38 MAPK but not JNK in the Nrf2-dependent modulation of DC immune functions.

##### Loss of Nrf2 Leads to Increased Basal Phosphorylation of CREB and ATF1 Transcription Factors in Immature DCs

**FIGURE 4.**
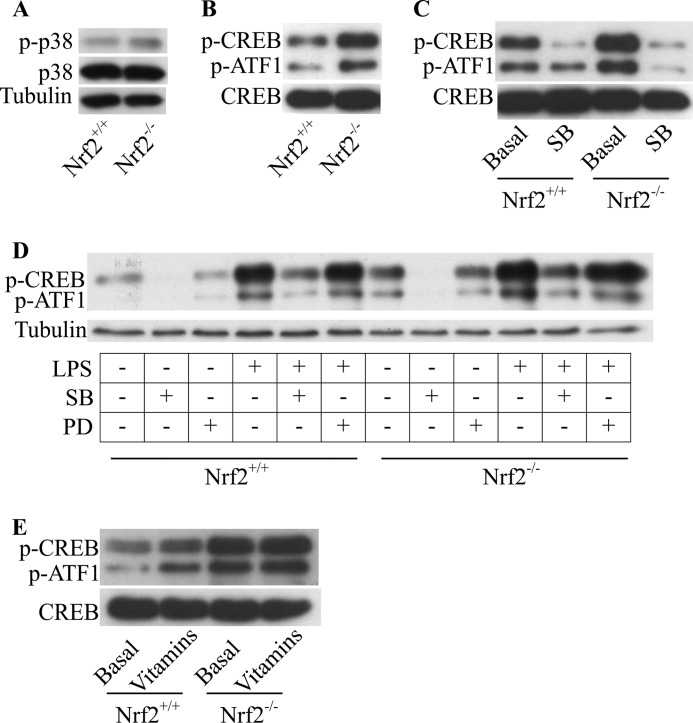
**Loss of Nrf2 perturbs p38 MAPK-CREB/ATF1 signaling in iDCs.** Whole cell lysates from Nrf2^+/+^ and Nrf2^−/−^ iDCs were subjected to SDS-PAGE. Western immunoblotting was used to determine the levels of phospho-p38 (p-p38) total p38 (p38), and tubulin (*A*). *B*, phospho-CREB (p-CREB), phospho-ATF1 (p-ATF1), and total CREB (CREB). *C*, whole cell lysates from Nrf2^+/+^and Nrf2^−/−^ iDCs treated with or without 20 μm of p38 activity inhibitor SB203580 (*SB*) for 1 h were subjected to SDS-PAGE. Phosphorylation of CREB and ATF1 (p-CREB and p-ATF1) and total CREB (CREB) were assessed. *D*, whole cell lysates from Nrf2^+/+^ and Nrf2^−/−^ iDCs treated with or without 50 μm of ERK1/2 inhibitor, PD98059 (*PD*), or 20 μm of p38 MAPK inhibitor, SB203580 (*SB*) for 1 h in the presence or absence of LPS (1 μg/ml) for the last 30 min were subjected to SDS-PAGE. Phosphorylation of CREB and ATF1 (p-CREB and p-ATF1) and total tubulin (as loading control) were assessed. *E*, whole cell lysates from Nrf2^+/+^ and Nrf2^−/−^ iDCs treated with or without vitamins C (1 mm) and E (100 μm) for 48 h were subjected to SDS-PAGE. Phosphorylation of CREB (p-CREB) and ATF1 (p-ATF1) and total CREB (CREB) were assessed. Data are representative of three independent experiments.

As the activity of p38 MAPK is regulated by phosphorylation, we assessed the phosphorylation state of p38 MAPK. Western blotting analysis revealed marginal differences in p38 MAPK phosphorylation between Nrf2^−/−^ and Nrf2^+/+^ iDCs (Fig. 4*A*). Major downstream effectors of p38 MAPK are the transcription factors, CREB and ATF1. Serine phosphorylation of CREB and ATF1 by upstream MAPKs is required for their activation. We measured the phosphorylation state of CREB and ATF1 and observed that CREB and ATF1 were hyperphosphorylated under basal conditions in Nrf2^−/−^ iDCs (Fig. 4*B*). A significant reduction in the phosphorylation of CREB and ATF1 was observed upon inhibiting p38 MAPK activity using a pharmacological inhibitor (SB203580) as seen in Fig. 4*C*. This suggests that CREB and ATF1 phosphorylation in DCs are dependent on p38 MAPK activity. There is evidence in other cell types that in addition to p38 MAPK, ERK1/2 can also mediate CREB/ATF1 phosphorylation. We tested the requirement of ERK1/2 for phosphorylation of CREB and ATF1 in the DCs under basal and LPS-stimulated conditions using an ERK1/2 inhibitor (PD98059). As seen in Fig. 4*D*, inhibition of ERK1/2 did not reduce the level of phospho-CREB/ATF1 under basal conditions. LPS stimulation markedly increased the phosphorylation of CREB/ATF1 in Nrf2^+/+^ and Nrf2^−/−^ iDCs (Fig. 4*D*). Inhibition of ERK1/2 did not reduce the phosphorylation of CREB/ATF1 under LPS-stimulated conditions in both Nrf2^+/+^ and Nrf2^−/−^ iDCs (Fig. 4*D*). Consistent with the previous result (Fig. 4*C*), p38 MAPK inhibition reduced CREB/ATF1 phosphorylation levels both basally and upon LPS stimulation. To test whether the basal hyperphosphorylation of CREB/ATF1 in Nrf2^−/−^ iDCs could be the result of elevated ROS (36), we treated the DCs with vitamins and show that decreasing elevated ROS did not reverse the increased CREB/ATF1 phosphorylation (Fig. 4*E*). These results indicate that Nrf2 is required for controlling the activity of the p38 MAPK-CREB/ATF1 signaling axis in DCs.

##### Loss of Nrf2 Leads to the Dysregulated IL-10 Production in DCs

**FIGURE 5.**
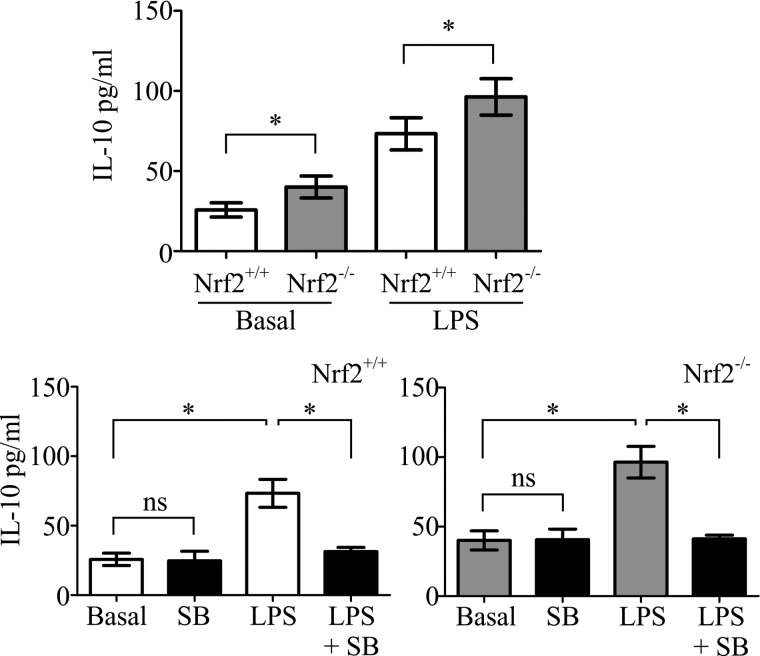
**Loss of Nrf2 results in elevated IL-10 production by DCs.** Nrf2^+/+^ and Nrf2^−/−^ DCs were incubated with or without 20 μm p38 MAPK inhibitor, SB203580 (*SB*) and/or LPS (1 μg/ml) for 48 h. Levels of IL-10 in supernatants were measured by ELISA. Data derived from two independent experiments are presented as average pg/ml ± S.D. Statistical significance was tested by one-way ANOVA (*, *p* < 0.05; *NS*, not significant).

Transcription of the anti-inflammatory cytokine IL-10 is regulated by CREB/ATF1 activity (37). We therefore measured the levels of IL-10 secreted by the iDCs. As shown in Fig. 5, Nrf2^−/−^ iDCs produce higher levels of IL-10 in comparison to Nrf2^+/+^ DCs under basal conditions (40.1 pg/ml *versus* 25.7 pg/ml *p* < 0.05). Furthermore, upon LPS stimulation, Nrf2^−/−^ iDCs produced levels of IL-10, which was greater than that produced by LPS-stimulated Nrf2^+/+^ iDCs (96.3 pg/ml *versus* 73.3 pg/ml *p* < 0.05). Although basal production of IL-10 was not sensitive to p38 MAPK inhibition, a significant reduction in LPS-induced IL-10 production (Nrf2^+/+^, 73.3 pg/ml to 31.3 pg/ml, *p* < 0.05; Nrf2^−/−^, 96.3 pg/ml to 41.2 pg/ml, *p* < 0.05) was observed in both Nrf2^+/+^ and Nrf2^−/−^iDCs treated with SB203580 (Fig. 5). This result suggests that LPS-stimulated but not basal IL-10 production in iDCs is dependent on p38 MAPK-CREB activity. Taken together, our findings suggest that the p38 MAPK-CREB/ATF1 signaling axis contributes to the Nrf2-mediated regulation of DC immune function.

##### Inhibition of HO-1 Activity Leads to Increased DC Co-stimulatory Receptor Expression and Hyperphosphorylation of CREB/ATF1

**FIGURE 6.**
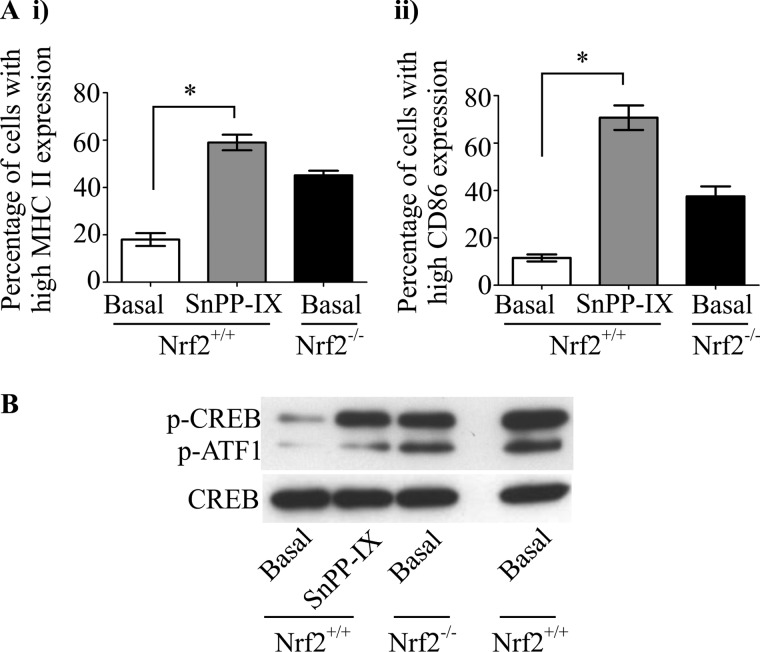
**HO-1 activity modulates DC phenotype and CREB/ATF phosphorylation.** Nrf2^+/+^ iDCs were treated with or without tin protoporphyrin IX dichloride (*SnPP-IX*, 5 μm) for 14 h. *A*, expression of MHC II (*i*) and CD86 (*ii*) was determined by flow cytometry and presented as percentage of cells expressing high MHC II or CD86. Data derived from three independent experiments are presented as average percentage ± S.D. Statistical significance was assessed using unpaired Student's *t* test (*, *p* < 0.05). *B*, whole cell lysates from Nrf2^+/+^ iDCs treated with or without tin protoporphyrin IX dichloride (5 μm) for 2 h and from untreated Nrf2^−/−^ iDCs were subjected to SDS-PAGE and phosphorylation status of CREB and ATF1 (p-CREB and p-ATF1) assessed by Western blotting. Lysate from Nrf2^+/+^ iDCs treated with LPS (1 μg/ml, 30 min) was used as positive control. Total CREB was assessed for equal loading of lanes.

A key Nrf2-transcribed gene involved in cellular homeostasis is HO-1. To test whether HO-1 is involved in Nrf2-mediated regulation of co-stimulatory molecule expression, Nrf2^+/+^ iDCs were treated with HO-1 inhibitor, tin protoporphyrin IX dichloride. As shown in Fig. 6*A*, tin protoporphyrin IX dichloride treatment increases cell surface expression of MHC II and CD86 in Nrf2^+/+^ iDCs compared with untreated Nrf2^+/+^ iDCs (MHC II 59.2% *versus* 18.1%, *p* < 0.05; CD86 70.7% *versus* 11.5%, *p* < 0.05). In addition, inhibition of HO-1 activity was shown to increase the phosphorylation of CREB/ATF1 in Nrf2^+/+^ iDCs (Fig. 6*B*). This observation suggests that one of the mechanisms by which Nrf2 modulates co-stimulatory molecule expression and CREB/ATF phosphorylation is through its effect on HO-1 function.

## DISCUSSION

Understanding the role of Nrf2 in DC biology requires the definition of critical molecular pathways that are subject to modulation by Nrf2 activity. Nrf2 is central to redox homeostasis, and it has been shown that in the absence of Nrf2, there is elevated ROS in DCs (14, 26). Although there is evidence suggesting that increased ROS is associated with elevated co-stimulatory changes (38, 39), our results demonstrate that in the context of Nrf2 deficiency, ROS does not directly underlie these changes in immature DCs. A possible explanation for this is that increased co-stimulatory molecule expression in response to physiological stimuli is usually due to transient and not persistent elevation of ROS (40). Sustained elevations of ROS levels as seen in Nrf2^−/−^ iDCs are therefore likely to result in cellular adaptive changes that cannot be simply reversed by rebalancing ROS levels but require more complex cellular reprogramming (41, 42). Our findings suggest that Nrf2-mediated regulation of DC function is not solely dependent on its role in redox homeostasis. This is consistent with our previous results wherein lowering the levels of the redox-regulating molecule, GSH in Nrf2^+/+^ iDCs does not recapitulate the altered phenotype and function of Nrf2^−/−^ iDCs.

Enhanced Nrf2 activity through the use of sulforaphane has been shown to suppress p38 MAPK pathway in endothelial cells (43). Our observations from this and an earlier report (26) also suggest that the p38 but not ERK1/2 or JNK is the main MAPK that is involved in Nrf2-mediated regulation of immature DC function. The inhibition of p38 MAPK causes a marked but incomplete reversal of DC function in Nrf2-deficient DCs, indicating that other pathways or factors are also involved. Indeed, histone deacetylases have also been implicated in the Nrf2-associated changes in phenotype (26).

The downstream target of p38 MAPK, CREB, has been associated with the regulation of key DC immune functions, including the expression of co-stimulatory molecules (8). Our finding of constitutive hyperphosphorylation of CREB and ATF1 in Nrf2^−/−^ iDCs highlights the requirement of Nrf2 in maintaining the integrity of the p38 MAPK-CREB/ATF1 pathway in iDCs. Using pharmacological inhibitors, we confirmed that CREB and ATF1 hyperphosphorylation was selectively mediated by p38 MAPK and not ERK1/2. The other MAPK, JNK is not involved in CREB/ATF1 phosphorylation (44) and hence was not examined in this study. In keeping with the lack of involvement of ROS in altered co-stimulatory molecule expression, reducing ROS did not alter CREB/ATF1 phosphorylation levels. These observations confirm the selective involvement of p38-CREB/ATF1 axis in Nrf2-mediated regulation of DC function.

In addition to MAPKs, the phosphorylation state of CREB can be regulated by protein phosphatases (45). Key phosphatases that act on CREB and regulate its phosphorylation are PP1 (46) and PP2A (7). It is possible that changes in PP1 and/or PP2A activity could also contribute to the increased CREB phosphorylation in Nrf2^−/−^ iDCs. It will be interesting to measure PP1 and PP2A activity in the DCs to test this possibility.

There is evidence that stimuli-induced IL-10 production requires CREB/ATF1 activation (37). Surprisingly, we found that the basal IL-10 production could not be reduced by inhibition of the p38-CREB/ATF1 pathway. However, LPS-induced IL-10 secretion was sensitive to inhibition of this pathway. This suggests that there are differential requirements for p38-CREB/ATF1 in the transcriptional regulation of IL-10 under basal *versus* stimulated conditions. It is pertinent to note that although the level of CREB/ATF1 phosphorylation in Nrf2^−/−^ iDCs under basal conditions is comparable with that in LPS-stimulated iDCs, the amount of IL-10 secreted basally is only half of that in LPS-stimulated iDCs. This indicates that CREB/ATF1 phosphorylation alone is not sufficient for IL-10 synthesis. To test the direct functional consequence of increased CREB/ATF1 phosphorylation in Nrf2^−/−^ iDCs, other readouts of CREB/ATF1 function such as levels of Bcl2 (47) or degree of cell survival could be measured.

Transactivation by CREB/ATF1 requires its association with cofactors such as CREB binding protein (CBP) (48). There are data demonstrating that availability of cellular CBP is limited and that CBP is also utilized by other transcription factors, including Nrf2 and NF-κB (49). It is therefore suggested that Nrf2 and NF-κB (in addition to CREB) compete for the available CBP (50–52). In the absence of Nrf2, more CBP is available for use by NF-κB and CREB. This could partly explain the increased levels of a number of genes transcribed by these transcription factors in Nrf2^−/−^ iDCs (53, 54). A candidate gene product of Nrf2 transactivation that could potentially account for the influence of Nrf2 in DC function and intracellular signaling is HO-1. Our results indicate that HO-1 activity is required to maintain iDC phenotype and prevent basal hyperphosphorylation of CREB/ATF. Evidence implicating HO-1 in modulating DC maturation comes from studies that demonstrate inhibition of activation-induced DC maturation when HO-1 is overexpressed (25). The molecular mechanism/s through which HO-1 modulates CREB/ATF1 phosphorylation and DC maturation are unclear and is the focus of our ongoing investigations. Our data, in conjunction with other studies, suggest that Nrf2 activity can affect cell signaling both at the level of p38-CREB/ATF1 and at the level of transcriptional cofactors (*i.e.* CBP).

In summary, our data implicates p38 MAPK-CREB/ATF1 axis as a key signaling pathway that is regulated by Nrf2 in DCs. The molecules within this pathway, as well as HO-1, could represent targets for pharmacological intervention in disease states that arise from dysregulated Nrf2 function.

## Footnotes

3 The abbreviations used are:

DC: dendritic cell
Nrf2: nuclear factor-erythroid 2 (NF-E2) p45-related factor-2
iDC: immature DC
ROS: reactive oxygen species
NP68: nucleoprotein 68
CREB: cAMP response element-binding protein
ATF1: activating transcription factor 1
HO-1: Heme oxygenase-1
ANOVA: analysis of variance
CBP: CREB binding protein
PP1: protein phosphatase 1
PP2A: protein phosphatase 2A.
